# Central Texas Medical Society

**Published:** 1890-01

**Authors:** 


ON THE 14th instant, the famous Central Texas Medical Association held an interesting meeting at Waco. It wound up with an oyster supper. The JOURNAL acknowledges the courtesy of an invitation, and regrets much its inability to send a representative to participate in the delightful *menu* set out for the occasion.

---

OUR poet, after a few weeks' rest, sends the following:

"Lives of poor men oft remind us,
Honest toil don't stand a chance;
More we work, we leave behind us,
Bigger patches on our pants;
On our pants, once new and glossy,
Now are patches of a different hue,
Just because those who owe us,
Will not pay up what is due."

—La Veta Times.

